# Supplementary material for: Association between rare, genetic variants linked to autism and ultrasonography fetal anomalies in children with autism spectrum disorder
Source: J Neurodev Disord. 2024 Sep 30;16:55. doi: 10.1186/s11689-024-09573-6 (PMC11443733; doi:10.1186/s11689-024-09573-6)
Supplement: Supplementary file 1 — Supplementary Material 1 [file 11689_2024_9573_MOESM1_ESM.docx]

| **Supplementary Table S1.**  Anomalies Detected During Anatomy Fetal Survey | | | |
| --- | --- | --- | --- |
| **Other** | **Heart** | **Urinary System** | **Head & Brain** |
| Single Umbilical Artery (SM) | Echogenic Intracardiac Focus (SM) | Pyelectasis (SM) | Choroid Plexus Cyst (SM) |
| Persistent Right Umbilical Vein (SM) | Ventricular Septal Defect (SA) | Single Kidney (SA) | Microcephaly <3 SD) (SA) |
| Echogenic Bowel (SM) |  | Cystic Kidney (SA) | Macrocephaly (>3 SD) (SA) |
| Enlarged Fetal Stomach (SM) |  | Dual Collecting System (SA) | Ventriculomegaly (SA) |
|  |  |  | Mega Cisterna Magna (SA) |
| SM = Soft Marker, SA – Structural Anomaly | | | |
