# Supplementary material for: Association between rare, genetic variants linked to autism and ultrasonography fetal anomalies in children with autism spectrum disorder
Source: J Neurodev Disord. 2024 Sep 30;16:55. doi: 10.1186/s11689-024-09573-6 (PMC11443733; doi:10.1186/s11689-024-09573-6)
Supplement: Supplementary file 2 — Supplementary Material 2 [file 11689_2024_9573_MOESM2_ESM.docx]

| **Supplementary Table S2.** Clinical and sociodemographic characteristics of ASD children | | | |
| --- | --- | --- | --- |
| *P* value | Excluded  (n = 593) | Study cohort  (n = 126) | Variable |
| 0.279 ^a^ | 467(78.2) | 93(73.8) | Jewish, no. (%) |
| 0.317 ^a^ | 472(78.7) | 94(74.6) | Male, no. (%) |
| 0.090 ^a^ | 152(33.9) | 32(29.1) | Special education setting, no. (%) |
| 0.387 ^b^ | 31.0±6.1 | 30.4±6.0 | Maternal age, mean±SD, years |
| 0.241 ^b^ | 34.3±7.6 | 33.4±7.7 | Paternal age, mean±SD, years |
| **<0.001** **^b^** | 3.3±1.5 | 2.8±1.3 | Diagnosis age, mean±SD, years |
| 0.703 ^b^ | 76.1±16.6 | 77.0±16.4 | Cognitive score (IQ), mean±SD |
| **0.023 ^c^** | 7(6-9) | 8(6-9) | ADOS Comparison Score, median (IQR) |
| ^a^ Chi-square; ^b^ Two-sided t-test; ^c^ Mann-Whitney U test  Boldface type indicates statistically significant aOR at α<0.05. | | | |
