# Supplementary material for: Association between rare, genetic variants linked to autism and ultrasonography fetal anomalies in children with autism spectrum disorder
Source: J Neurodev Disord. 2024 Sep 30;16:55. doi: 10.1186/s11689-024-09573-6 (PMC11443733; doi:10.1186/s11689-024-09573-6)
Supplement: Supplementary file 4 — Supplementary Material 4 [file 11689_2024_9573_MOESM4_ESM.docx]

| **Supplementary Table S4.** Genetic characteristics of ASD children | | | |
| --- | --- | --- | --- |
| *P* value | Children without  ultrasound data  (n = 121) ^a^ | Children with ultrasound data  (n = 126)^a^ | Variable |
| 0.833 ^b^ | 56(46.3) | 60(47.6) | Any mutation |
| 0.414 ^b^ | 41(33.9) | 49(38.9) | Dominant |
| 0.917 ^b^ | 12(9.9) | 13(10.3) | De novo |
| 0.175 ^b^ | 7(5.8) | 3(2.4) | Recessive |
| 0.490 ^c^ | 1(0.8) | 0(0.0) | X-linked |
| 0.814 ^b^ | 30(2.9) | 34(27.2) | LoF |
| ^a^ Values are no. (%);  ^b^ Chi-square; ^c^ Fisher exact test | | | |
